# Supplementary material for: Accelerating 3D genomics data analysis with Microcket
Source: Commun Biol. 2024 Jun 1;7:675. doi: 10.1038/s42003-024-06382-4 (PMC11144199; doi:10.1038/s42003-024-06382-4)
Supplement: Supplementary file 2 — Supplementary Information [file 42003_2024_6382_MOESM2_ESM.pdf]

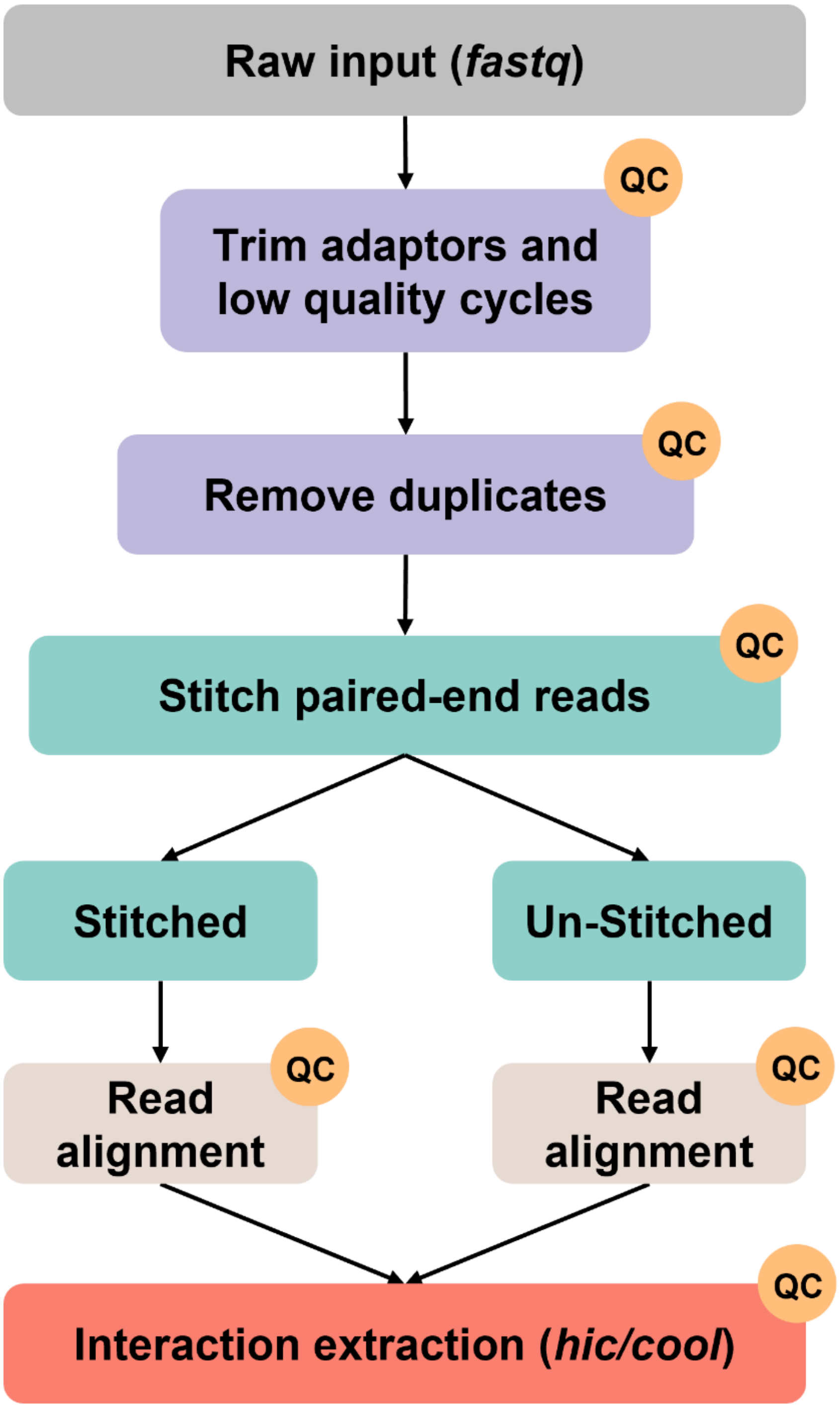

Figure S1. Schematic workflow of Microcket. QC: quality control; *fastq*, *pairs*, *hic*, and *cool* are file formats.

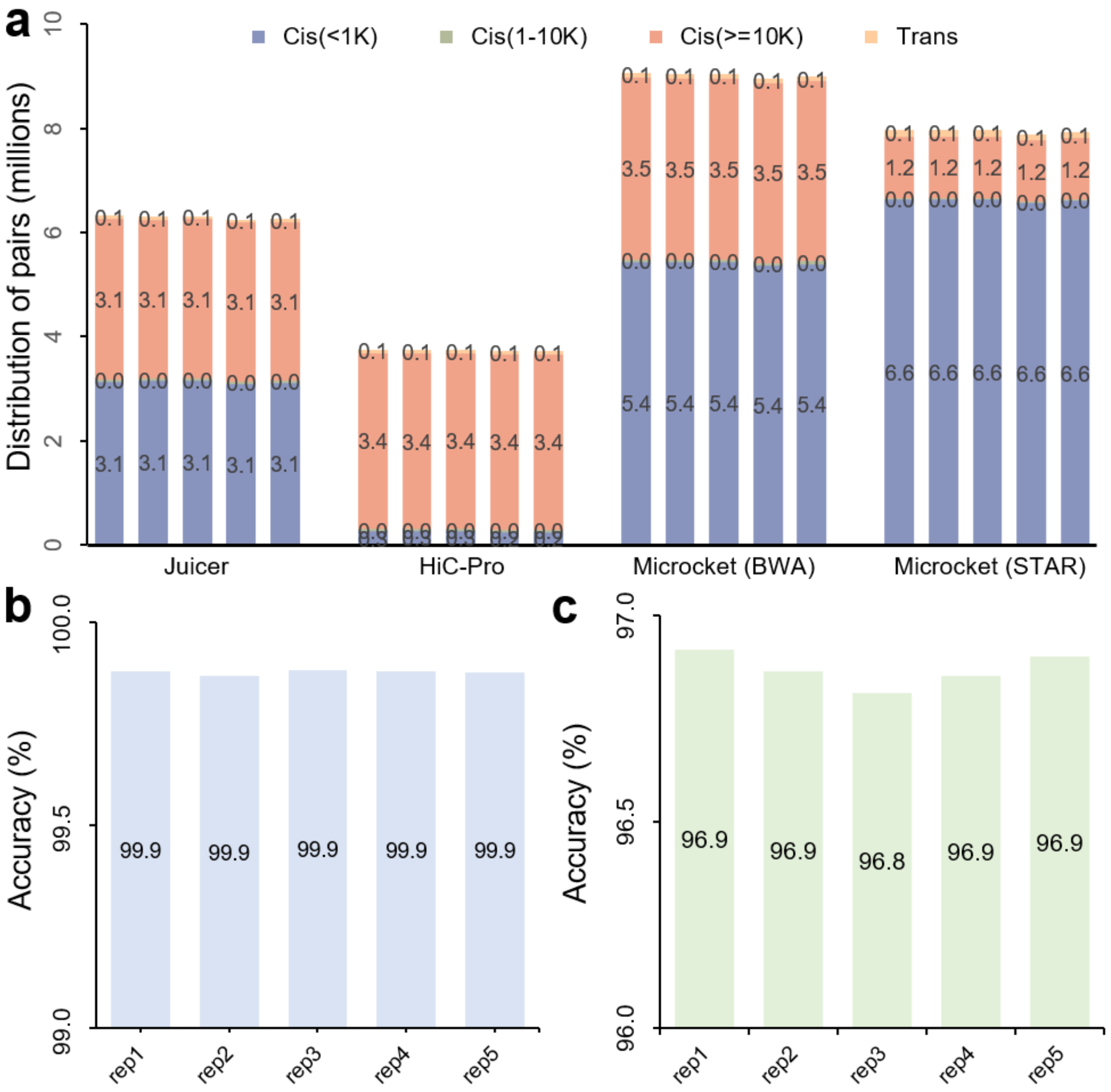

Figure S2. Analysis results on the *in silico* simulated 10 million reads. a) Distribution of reported pairs among different software; b) accuracy of pairs that were reported by Microcket with BWA and c) with STAR while not in Juicer.

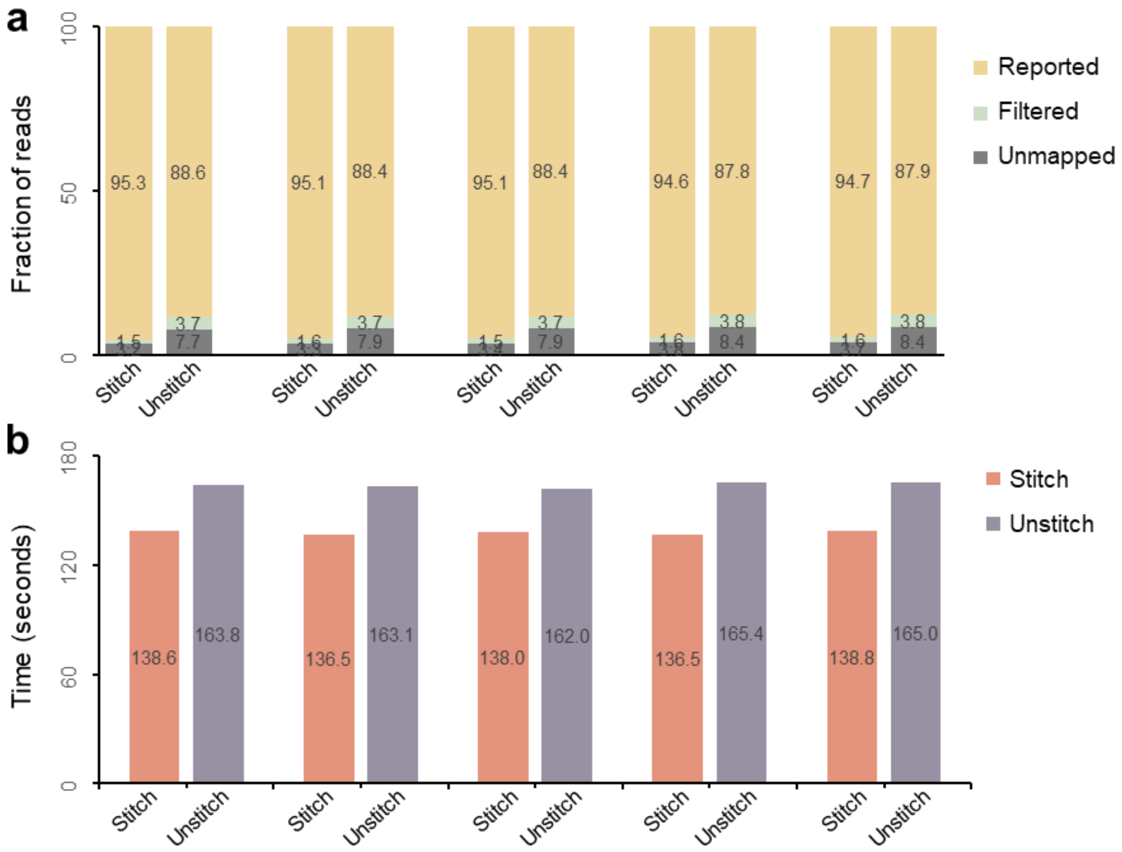

Figure S3. Evaluation of the read stitching stretegy on the *in silico* simulated reads. a) Distribution of analyzing results, and b) running time when the stitchable reads were analyzed with or without stitching.

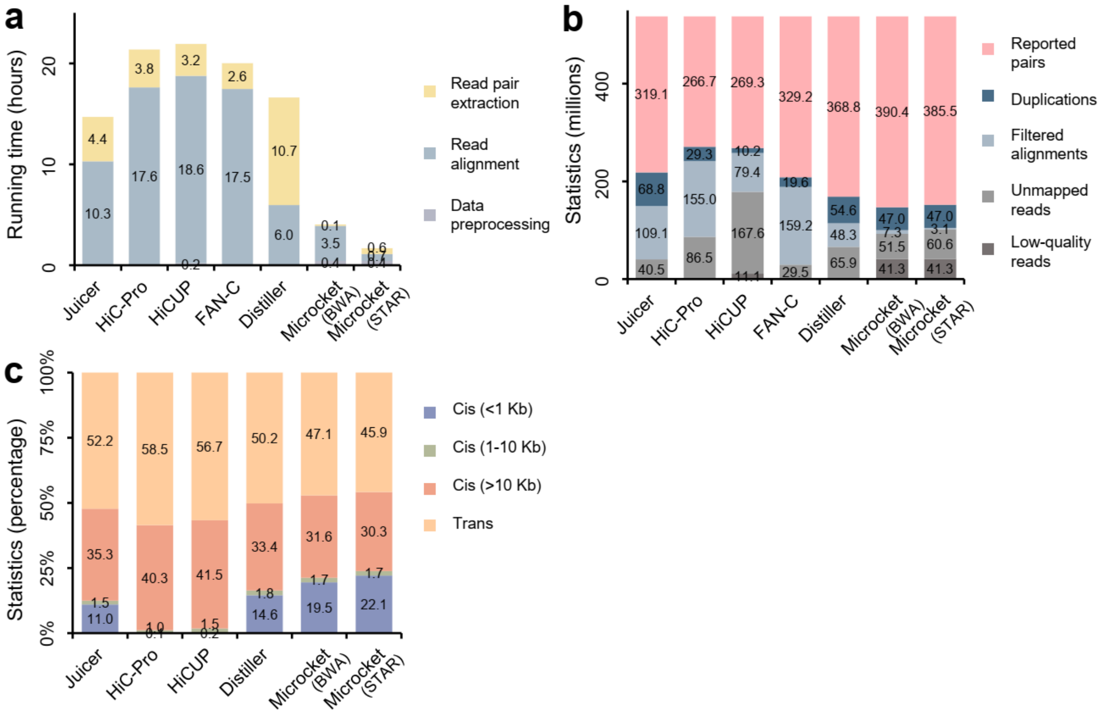

Figure S4. Benchmark evaluation results of Microcket versus the current tools on GM12878 Hi-C data in Dataset 1. a) Running time (in hours; averaged from 5 replicated runs); b) key statistics of the analysis (numbers were in millions); c) distributions of pairs reported by Juicer and Microcket (using BWA as the aligner).

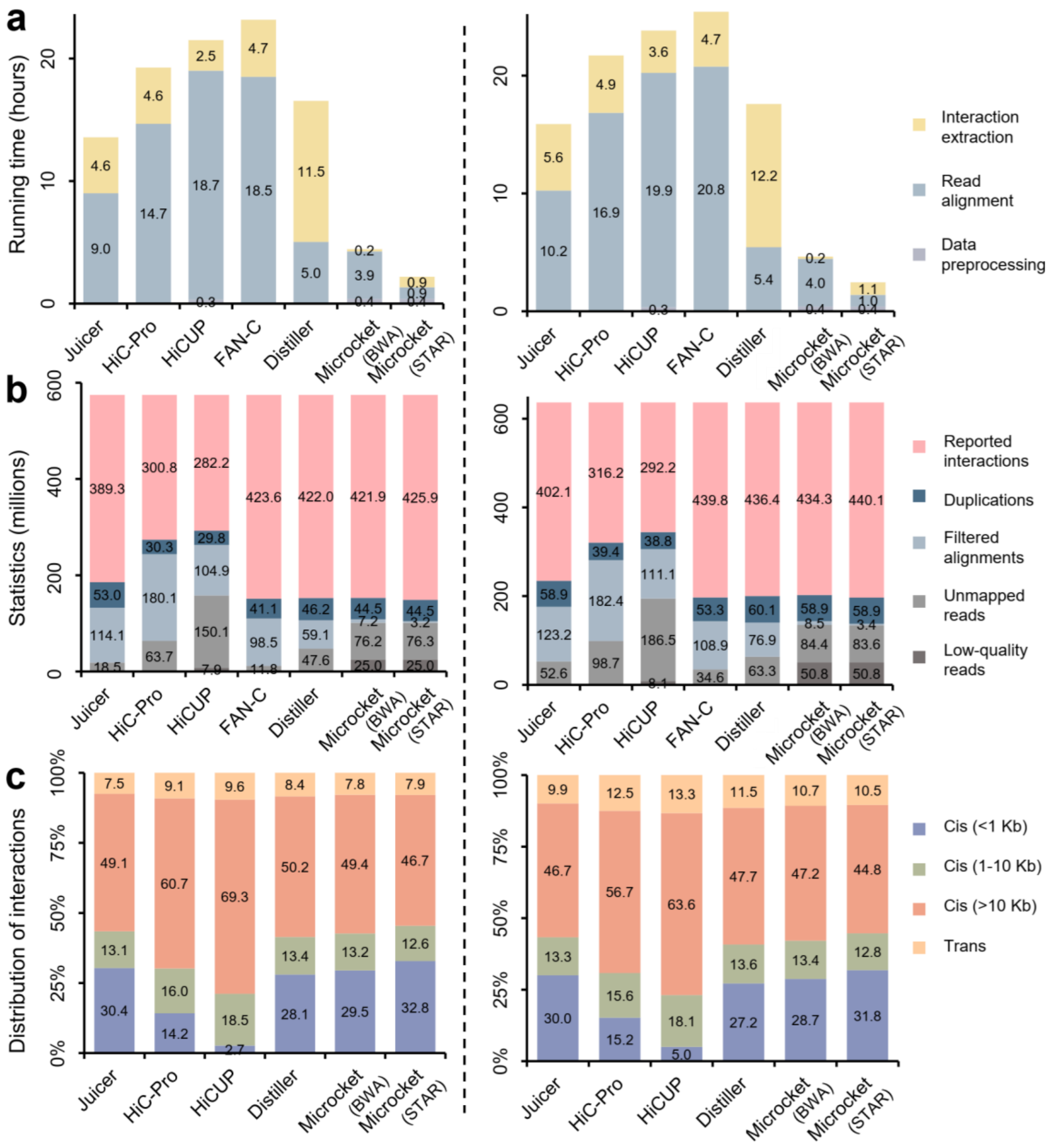

Figure S5. Benchmark evaluation results of Microcket versus the current tools on Hi-C data from LS147T (left) and SW480 (right) cell lines in Dataset 2. a) Running time (in hours; averaged from 5 replicated runs); b) key statistics of the analysis (numbers were in millions); c) distributions of pairs reported by Juicer and Microcket (using BWA as the aligner).

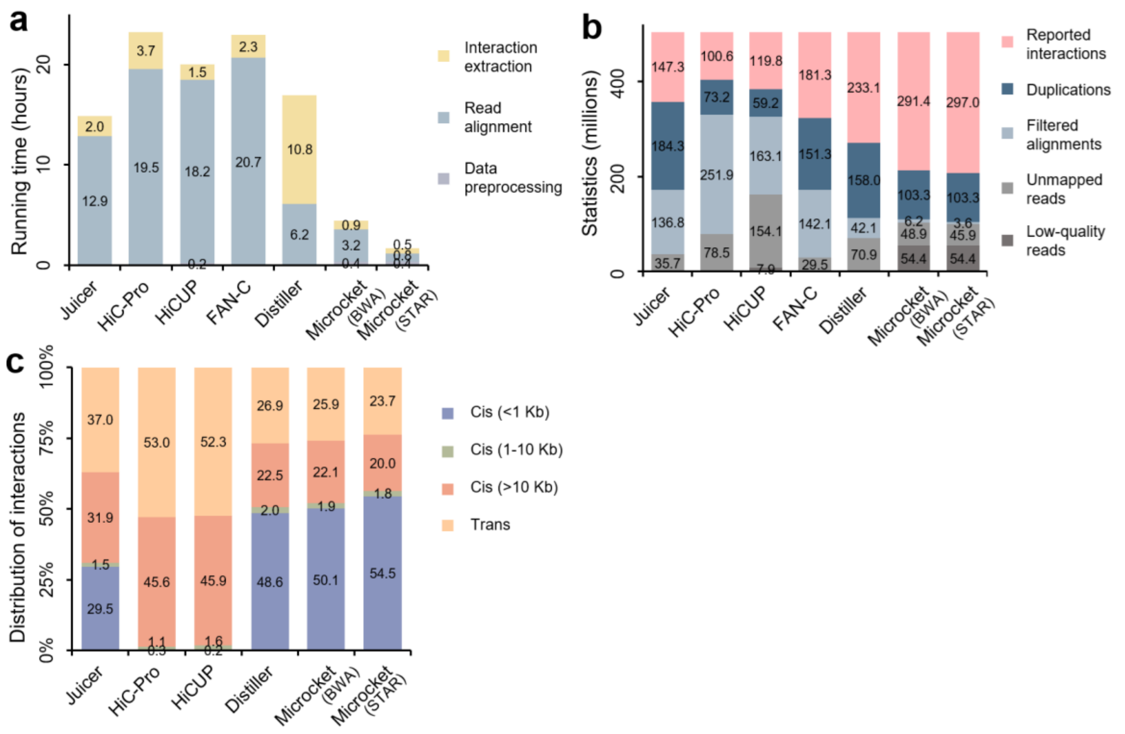

Figure S6. Benchmark evaluation results of Microcket versus the current tools on Hi-C data from liver tissue in Dataset 3. a) Running time (in hours; averaged from 5 replicated runs); b) key statistics of the analysis (numbers were in millions); c) distributions of pairs reported by Juicer and Microcket (using BWA as the aligner).

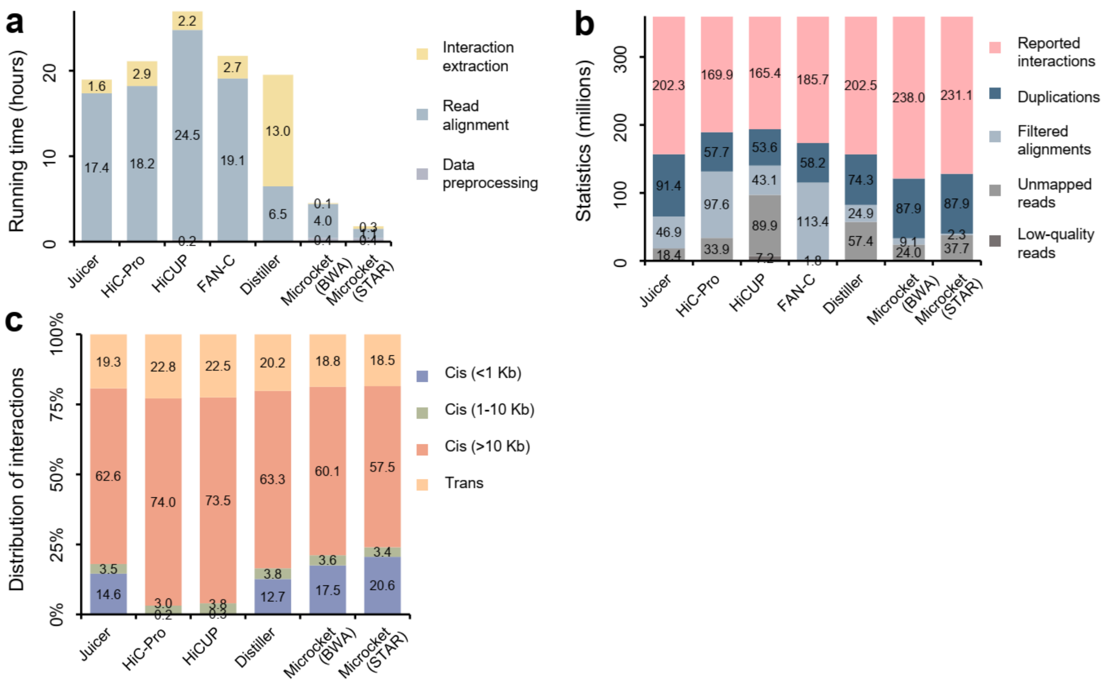

Figure S7. Benchmark evaluation results of Microcket versus the current tools on Hi-C data from astrocytes in Dataset 4. a) Running time (in hours; averaged from 5 replicated runs); b) key statistics of the analysis (numbers were in millions); c) distributions of pairs reported by Juicer and Microcket (using BWA as the aligner).

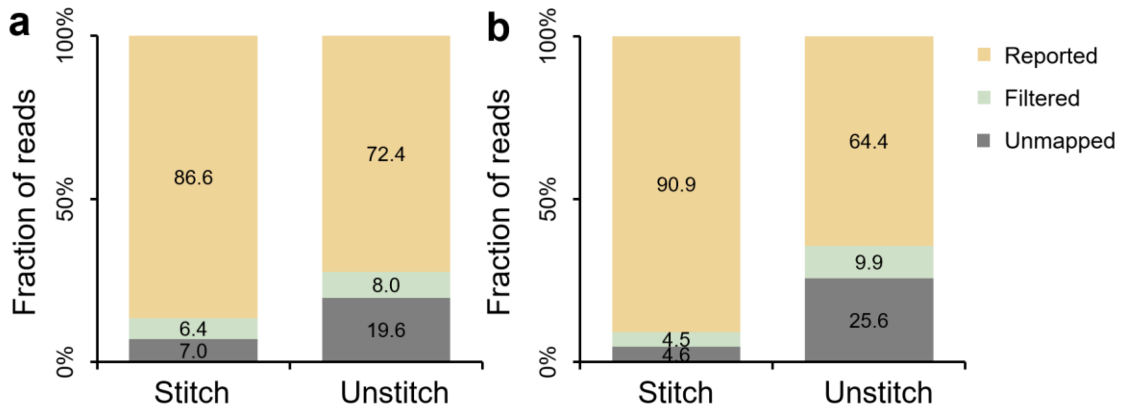

Figure S8. Distribution of analyzing results for the stitchable and unstitchable reads in Dataset 4. a) Neuron, and b) astrocyte cells.

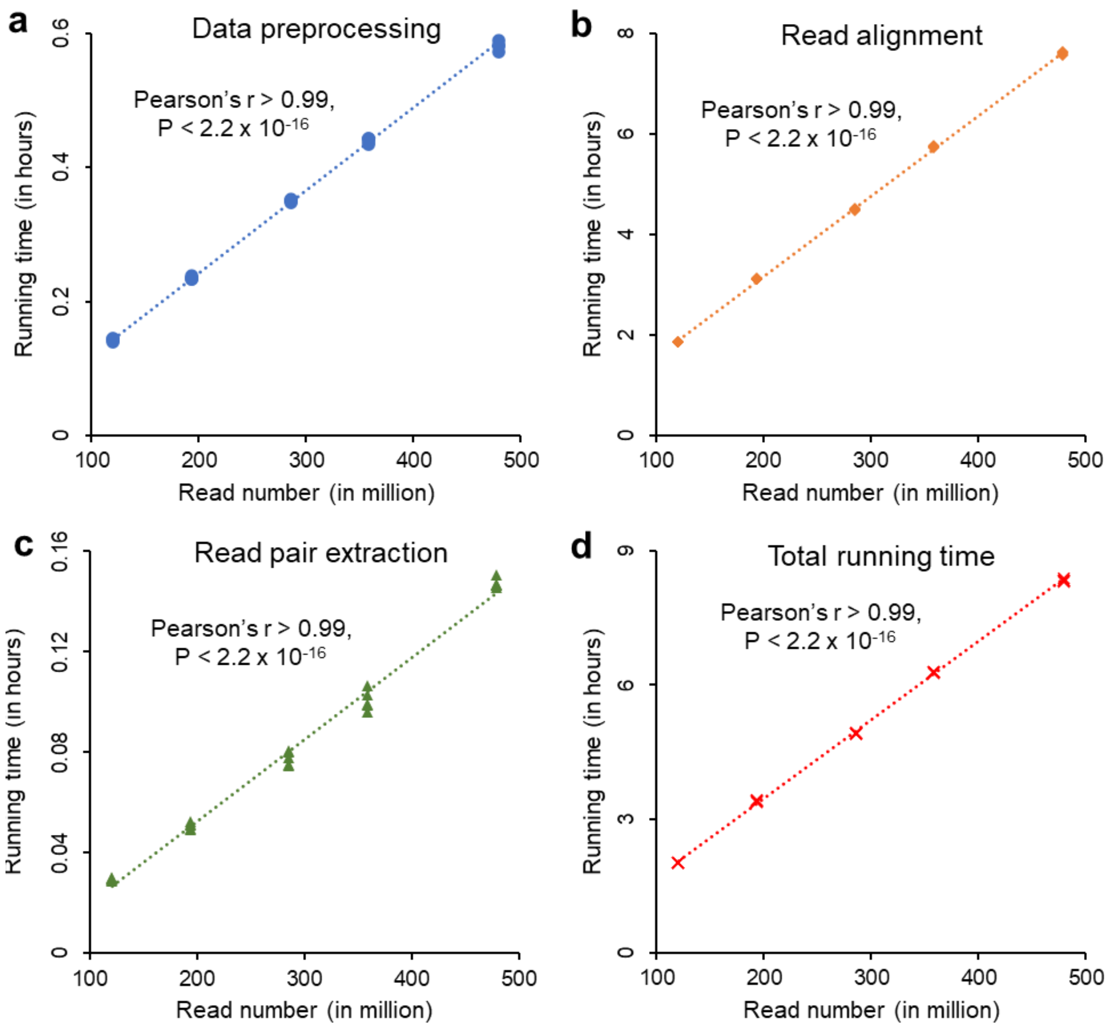

Figure S9. Relationship of Microcket's running time and sequencing read number. The Hi-C data from neuron in Dataset 4 was down-sampled to various sequencing depths for evaluation. a) Read preprocessing, b) alignment, c) pair extraction, and d) overall running time. Each experiment was repeated 5 times. P-values were calculated using linear regressions.

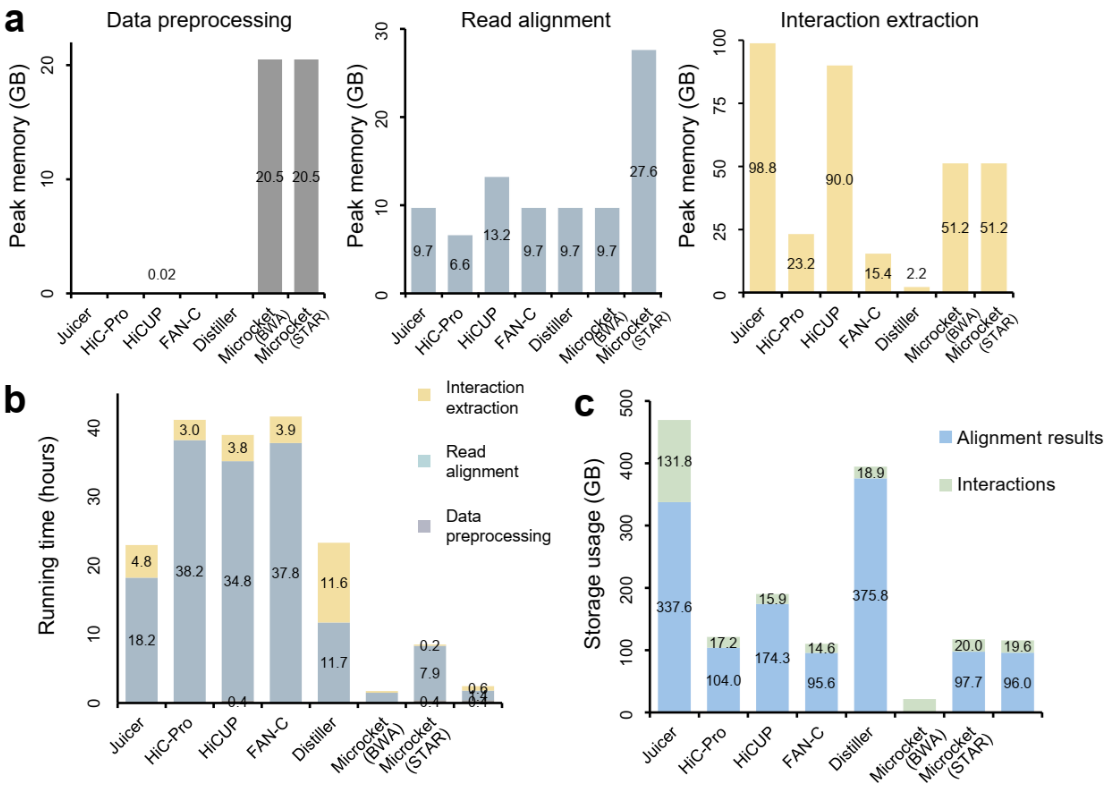

Figure S10. Benchmark evaluation results of Microcket versus the current tools on Hi-C data from IMR90 cell line in Dataset 1. a) Memory, and b) storage usage using 16-threads. c) Running time using 8-threads. Note that in the interaction-extraction step, Juicer, HiC-Pro, and Microcket tried to allocate high memory to speed up for systems with rich resources.

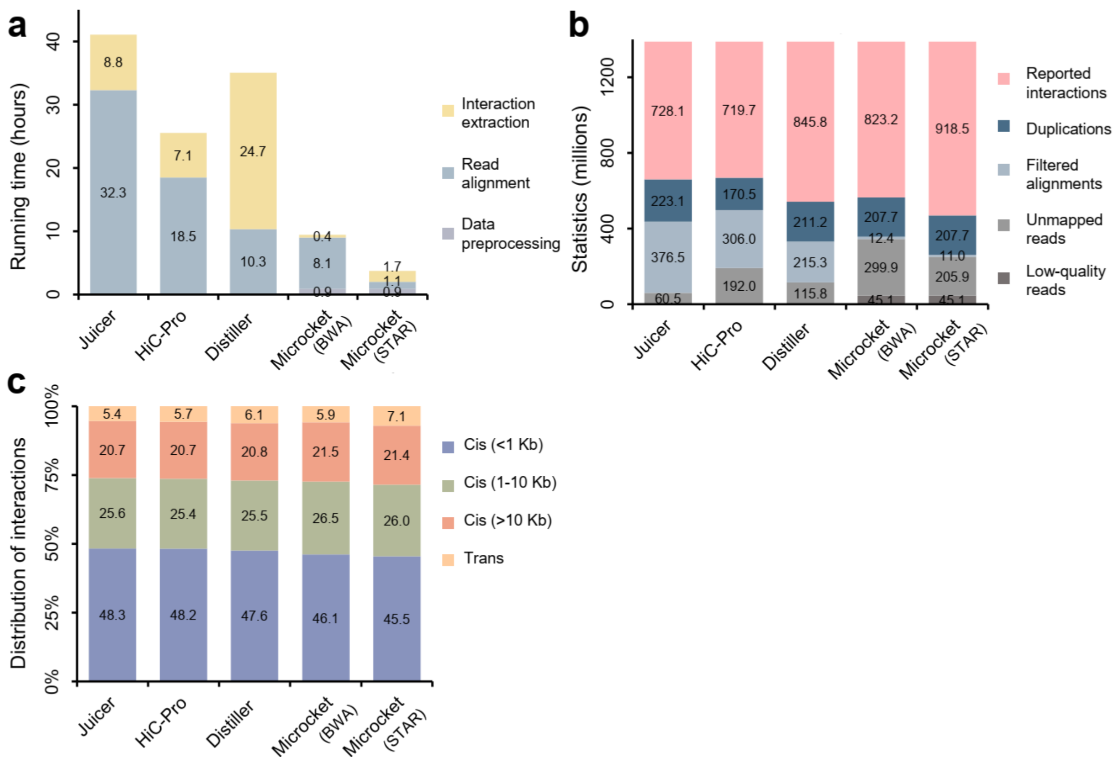

Figure S11. Benchmark evaluation results of Microcket versus the current tools on Micro-C data from HFFc6 cell line in Dataset 5. a) Running time (in hours; averaged from 5 replicated runs); b) key statistics of the analysis (numbers were in millions); c) distributions of pairs reported by Juicer and Microcket (using BWA as the aligner).

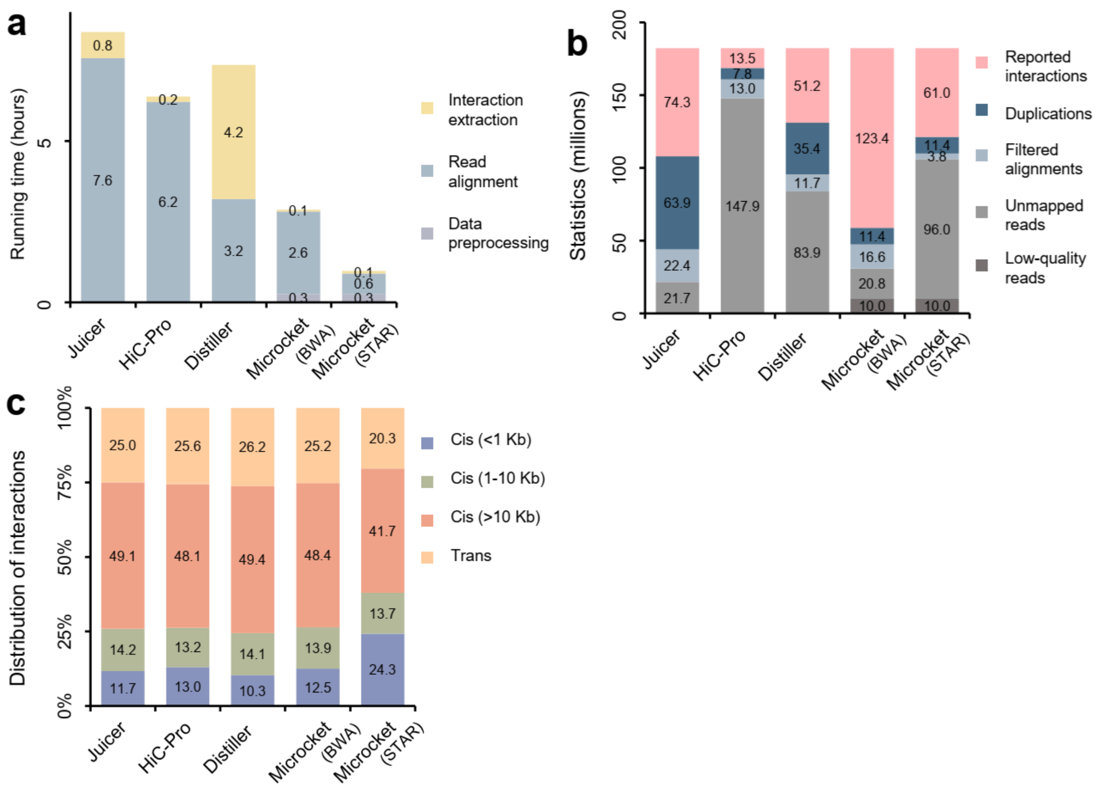

Figure S12. Benchmark evaluation results of Microcket versus the current tools on Micro-C data in Dataset 6. a) Running time (in hours; averaged from 5 replicated runs); b) key statistics of the analysis (numbers were in millions); c) distributions of pairs reported by Juicer and Microcket (using BWA as the aligner).

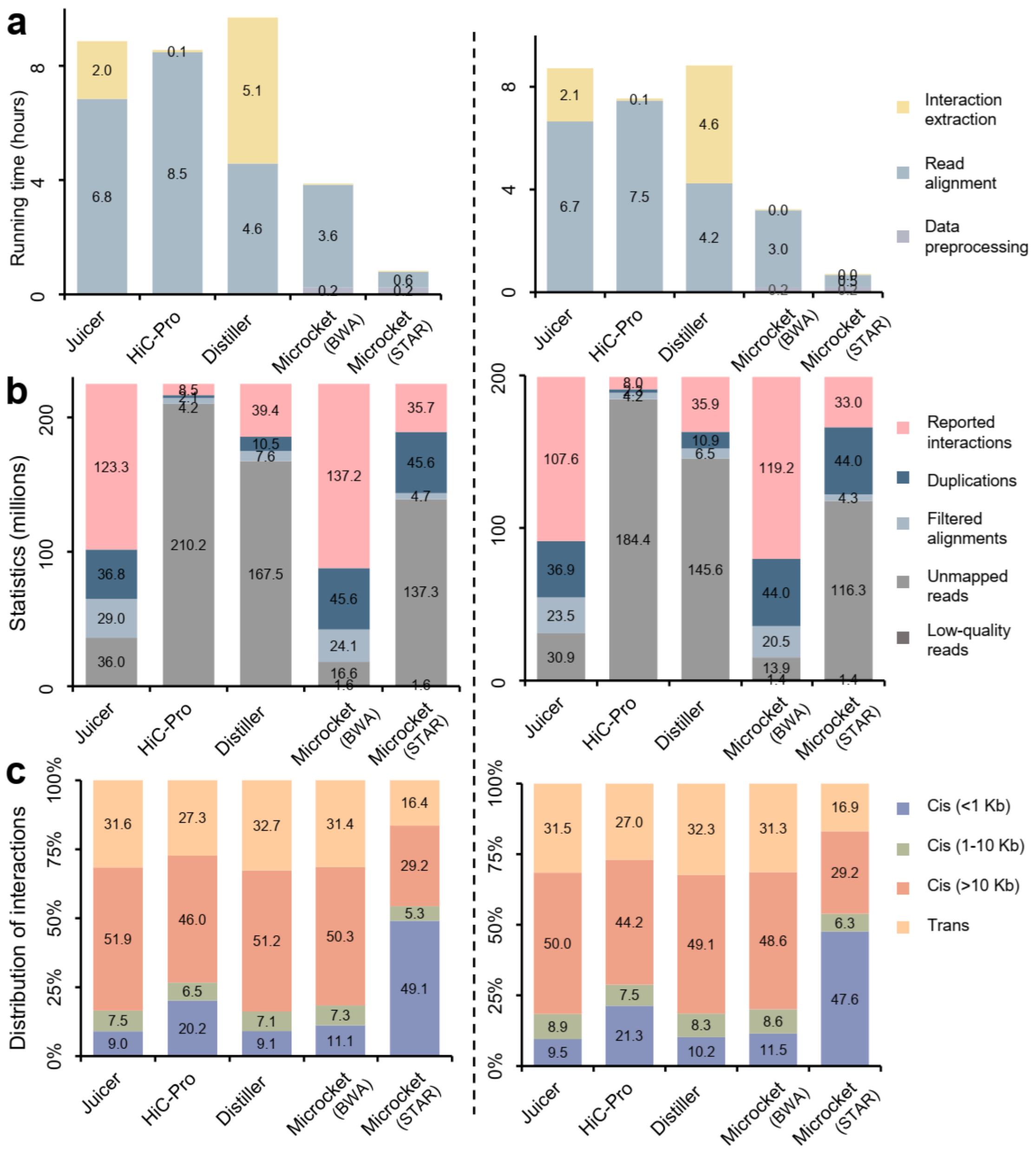

Figure S13. Benchmark evaluation results of Microcket versus the current tools on Micro-C data from ATRX- (left) and ATRX+ (right) cell lines in Dataset 7. a) Running time (in hours; averaged from 5 replicated runs); b) key statistics of the analysis (numbers were in millions); c) distributions of pairs reported by Juicer and Microcket (using BWA as the aligner).

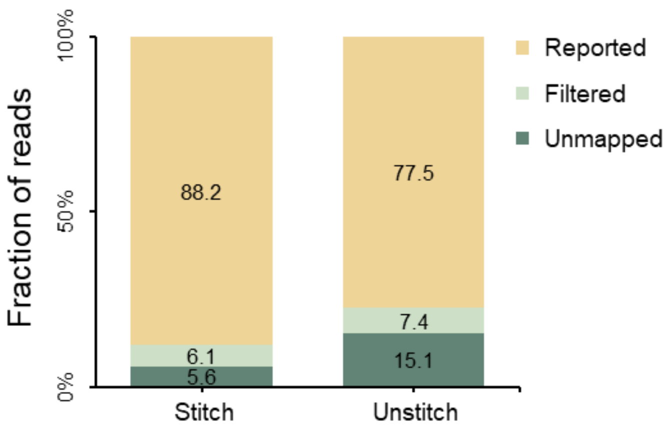

Figure S14. Distribution of analyzing results for the stitchable and unstitchable reads in Dataset 8.

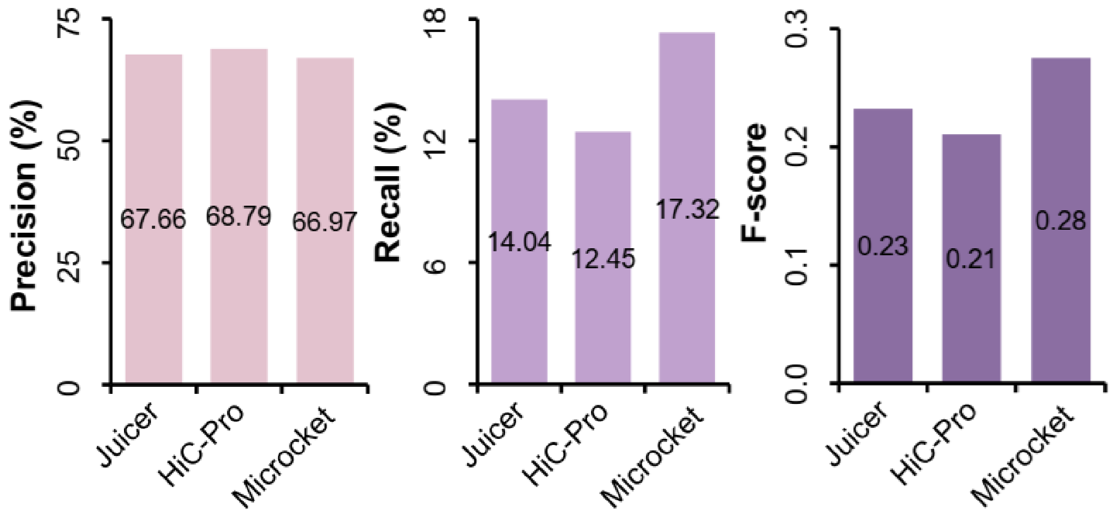

Figure S15. Performance evaluations of Microcket versus the current tools. We called and compared the loops using matrices generated by these tools on the Hi-C data from hESC cell line in Dataset 1 and 4DN's high-depth matrix (serving as "gold-standard").

Table S1. Proportion of stitchable reads in randomly selected datasets.

Protocol	Study ID	Run type	Accession number	Proportion of stitch-able reads (%)
Hi-C	SRP366243	PE150nt	SRR18500487	49.4
			SRR18500488	36.0
	SRP421771	PE150nt	SRR23520494	19.5
			SRR23520806	18.7
	SRP429367	PE151nt	SRR23973507	75.4
			SRR23973508	68.3
	SRP404349	PE150nt	SRR23618920	23.4
			SRR23618921	38.7
	SRP424859	PE151nt	SRR23636421	4.3
			SRR23636422	11.5
Micro-C	SRP322774	PE151nt	SRR14736881	85.9
			SRR14736882	82.2
	SRP324888	PE150nt	SRR22316008	34.3
			SRR22316012	20.7
	SRP377421	PE151nt	SRR19430685	24.1
			SRR19430686	23.8
	SRP387412	PE150nt	SRR20340598	56.1
			SRR20340610	54.4
	SRP395980	PE151nt	SRR21461209	52.5
			SRR21461211	56.2
