# Supplementary material for: Accelerating 3D genomics data analysis with Microcket
Source: Commun Biol. 2024 Jun 1;7:675. doi: 10.1038/s42003-024-06382-4 (PMC11144199; doi:10.1038/s42003-024-06382-4)
Supplement: Supplementary file 5 — Reporting summary [file 42003_2024_6382_MOESM5_ESM.pdf]

nature portfolio

Corresponding author(s): Kun Sun

Last updated by author(s): Apr 10, 2024

Reporting Summary

Nature Portfolio wishes to improve the reproducibility of the work that we publish. This form provides structure for consistency and transparency in reporting. For further information on Nature Portfolio policies, see our Editorial Policies and the Editorial Policy Checklist.

Statistics

For all statistical analyses, confirm that the following items are present in the figure legend, table legend, main text, or Methods section.

n/a	Confirmed	
☒	☐	The exact sample size (*n*) for each experimental group/condition, given as a discrete number and unit of measurement
☒	☐	A statement on whether measurements were taken from distinct samples or whether the same sample was measured repeatedly
☒	☐	The statistical test(s) used AND whether they are one- or two-sided<br>*Only common tests should be described solely by name; describe more complex techniques in the Methods section.*
☒	☐	A description of all covariates tested
☒	☐	A description of any assumptions or corrections, such as tests of normality and adjustment for multiple comparisons
☒	☐	A full description of the statistical parameters including central tendency (e.g. means) or other basic estimates (e.g. regression coefficient) AND variation (e.g. standard deviation) or associated estimates of uncertainty (e.g. confidence intervals)
☒	☐	For null hypothesis testing, the test statistic (e.g. *F*, *t*, *r*) with confidence intervals, effect sizes, degrees of freedom and *P* value noted<br>*Give P values as exact values whenever suitable.*
☒	☐	For Bayesian analysis, information on the choice of priors and Markov chain Monte Carlo settings
☒	☐	For hierarchical and complex designs, identification of the appropriate level for tests and full reporting of outcomes
☒	☐	Estimates of effect sizes (e.g. Cohen's *d*, Pearson's *r*), indicating how they were calculated

Our web collection on statistics for biologists contains articles on many of the points above.

Software and code

Policy information about availability of computer code

Data collection

Micro-C data for K562 cell line (dataset 8) has been deposited to Gene Expression Omnibus (GEO) under accession number GSE205500; Hi-C data for IMR90 and GM12878 cell lines (dataset 1) is available from GEO under accession numbers GSM1551583, GSM1551599, and GSM1551600; Hi-C data for HCT116, LS147T, and SW480 cell lines (dataset 2) is available from GEO under accession number GSE133928 (sample ids "HCT116-0h-1_HiC", "HCT116-0h-2_HiC", "LS174T-1_HiC", "LS174T-2_HiC", "SW480-1_HiC", and "SW480-2_HiC" were used in this study); Promoter-capture Hi-C data for human lung and liver tissue (dataset 3) is available from GEO under accession number GSE86189 (sample ids "STL001.LG1" and "STL011.LI11" were used in this study); Promoter-capture Hi-C data for neurons and astrocytes (dataset 4) is available from GEO under accession number GSE113481; Micro-C data for hESC and HFFc6 cell lines (dataset 5) is available from 4D Nucleosome Project under accession numbers: 4DNEXQMEU2O4, 4DNEXC1TYVLD, and 4DNEXXXWOYOB; preprocessed high-depth interaction matrices for hESC cell line is available from 4D Nucleosome Project under accession number 4DNFI2TK7L2F; Micro-C data for C42B cell line (dataset 6) is available from GEO under accession number GSE205000; Micro-C data for human fibroblasts (dataset 7) is available from GEO under accession number GSE212809.

Data analysis

During the benchmark evaluations, 16 threads were used for all software. The reference human genome version used was NCBI GRCh38 (hg38). For Distiller, default parameters were used; for HiC-Pro/HiCUP/FAN-C, the digestion-related parameters were manually set to match the experimental protocols of the corresponding datasets, while all the other parameters were kept default; for Microcket, default parameters were used except that '-b' option was set for K562 cell line and samples in dataset 3 to enable Microcket's built-in support for biological replicates (which was not natively supported by all HiC-Pro, HiCUP, FAN-C, and Distiller). Each analysis was repeated 5 times and the averaged running time was reported. For HiCUP, as the main pipeline stopped after alignment filtering, we used its utility program 'hicup2juicer' to obtain the interaction results. For FAN-C, as it did not report the fragment-level interaction pairs, distributions of the interactions and comparisons to Microcket were both omitted. To assess the concordances between Microcket and HiC-Pro/HiCUP/Distiller,

mapped reads with identical chromosomes and the distances between reported genomic coordinates < 200 bp (as the underline aligners used clipping and reported different endpoint for each fragment) were considered as consistent results.

For manuscripts utilizing custom algorithms or software that are central to the research but not yet described in published literature, software must be made available to editors and reviewers. We strongly encourage code deposition in a community repository (e.g. GitHub). See the Nature Portfolio guidelines for submitting code & software for further information.

Data

Policy information about availability of data

All manuscripts must include a data availability statement. This statement should provide the following information, where applicable:

- Accession codes, unique identifiers, or web links for publicly available datasets
- A description of any restrictions on data availability
- For clinical datasets or third party data, please ensure that the statement adheres to our policy

Micro-C data for K562 line has been deposited to Gene Expression Omnibus (GEO; accession code: GSE205500).

Human research participants

Policy information about studies involving human research participants and Sex and Gender in Research.

Reporting on sex and gender	Not relevant to this study.
Population characteristics	Not relevant to this study.
Recruitment	Not relevant to this study.
Ethics oversight	Not relevant to this study.

Note that full information on the approval of the study protocol must also be provided in the manuscript.

Field-specific reporting

Please select the one below that is the best fit for your research. If you are not sure, read the appropriate sections before making your selection.

☒ Life sciences ☐ Behavioural & social sciences ☐ Ecological, evolutionary & environmental sciences

For a reference copy of the document with all sections, see nature.com/documents/nr-reporting-summary-flat.pdf

Life sciences study design

All studies must disclose on these points even when the disclosure is negative.

Sample size	Not relevant to this study.
Data exclusions	Not relevant to this study.
Replication	Not relevant to this study.
Randomization	Not relevant to this study.
Blinding	Not relevant to this study.

Reporting for specific materials, systems and methods

We require information from authors about some types of materials, experimental systems and methods used in many studies. Here, indicate whether each material, system or method listed is relevant to your study. If you are not sure if a list item applies to your research, read the appropriate section before selecting a response.

Materials & experimental systems

n/a	Involved in the study
☒	☐ Antibodies
☒	☐ Eukaryotic cell lines
☒	☐ Palaeontology and archaeology
☒	☐ Animals and other organisms
☒	☐ Clinical data
☒	☐ Dual use research of concern

Methods

n/a	Involved in the study
☒	☐ ChIP-seq
☒	☐ Flow cytometry
☒	☐ MRI-based neuroimaging
